# Late presentation of unilateral lung agenesis in adulthood

**DOI:** 10.1186/s43055-021-00533-x

**Published:** 2021-06-22

**Authors:** Arshed Hussain Parry, Mujahed Abdulsattar Ibrahim Raheem, Hussam Hassan Ismail, Osama Sharaf

**Affiliations:** Department of Medical Imaging, King Hamad University Hospital, Al Sayh, Muharraq, Bahrain

**Keywords:** Lung agenesis, Pulmonary agenesis, Adulthood presentation, Opaque hemithorax, Case report

## Abstract

**Background:**

Pulmonary agenesis is a rare congenital anomaly with a reported prevalence of about 1 in 100,000 births. It may be bilateral or unilateral. Among the unilateral form, left lung agenesis is more common (70%); however, it is the right lung agenesis which carries a dismal prognosis due to the frequent association with a gamut of other congenital anomalies and greater degree of mediastinal shift leading to tracheo-bronchial and vascular distortion. The patients of unilateral pulmonary agenesis usually present in infancy or early childhood. Presentation in late adulthood as seen in our patient is rare. We present a case of left pulmonary agenesis that was diagnosed in 4th decade of life.

**Case presentation:**

A 36-year-old male presented with gradually progressive exertional dyspnea of 1 month duration. Clinical examination revealed tachycardia and tachypnea. Chest radiograph showed opaque left hemithorax with ipsilateral mediastinal shift. Computed tomography clinched the diagnosis by demonstrating absence of left main bronchus, lung and left pulmonary artery with shift of heart, and great mediastinal vessels into left hemithorax. The patient was managed conservatively and discharged with attachment to out-patient department for regular follow-up.

**Conclusion:**

Presentation of unilateral lung agenesis in late adulthood, as seen in the present case is extremely rare. This case report highlights that, a rare condition like unilateral pulmonary agenesis, should be considered in the list of differentials in an adult presenting with opaque hemithorax with ipsilateral mediastinal shift on radiography.

## Background

Pulmonary agenesis refers to the complete absence of pulmonary tissue, bronchi, and pulmonary vasculature, and is a rare congenital anomaly with a reported prevalence of about 1 in 100,000 births [1]. It may be bilateral or unilateral. The bilateral type is incompatible with extra-uterine existence. Among the unilateral form right lung agenesis carries a dismal prognosis due to the frequently associated cardiovascular anomalies and greater degree of mediastinal shift with resultant compression and distortion of mediastinal vessels and trachea [2]. These patients mostly present in neonatal period or early childhood with respiratory distress, recurrent chest infections, and feeding difficulties. More than half of the cases die in the first 5 years of life [3]. However, very rarely the condition may present in adulthood or may remain asymptomatic throughout the life [4].

## Case presentation

A 36-year-old male presented to our emergency department with history of exertional shortness of breath for last 1 month. The dyspnea progressively increased especially in the last 1 week. Patient gets dyspneic on climbing a flight of stairs and gets tired while carrying heavy objects, activities which would previously not make him dyspneic or tired. There was no history of cough or fever. Patient denied any history of chest pain, palpations, dizziness, orthopnea, or lower limb swelling. No history of contact with COVID-19 cases was forthcoming. Past history was insignificant. On examination, patient was conscious, alert, oriented, and afebrile. Heart rate was 98 beats per minute and breathing rate was 28 breaths per minute. Airway was patent and patient was breathing adequately. No signs of circulatory compromise were seen.

**Fig. 1:**
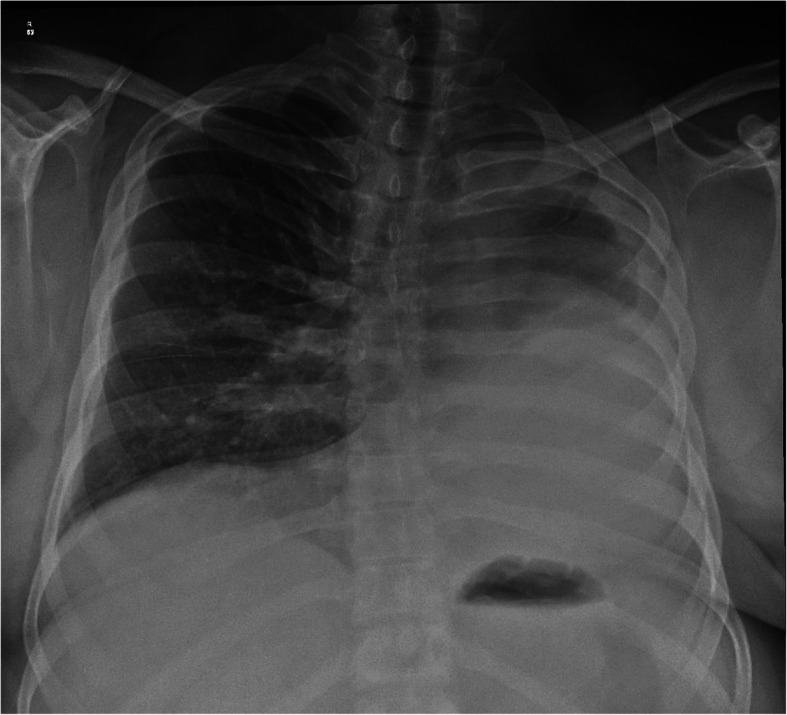
Chest radiograph in frontal projection showing homogenous opacity of left hemithorax with mediastinal shift and contralateral herniation of right lung into left upper hemithorax

Examination of chest revealed reduced chest excursions, absent air entry, and breath sounds on left side of chest. Chest radiograph revealed near total opacification of left hemi-thorax particularly affecting the mid and lower zones. Heart and mediastinal structures were shifted toward left side with hyperexpanded right lung crossing across the midline to the left upper hemithorax (Fig. 1).

Owing to the clinical findings of tachycardia and tachypnea, pulmonary embolism was suspected and computed tomographic pulmonary angiography (CTPA) was requested.

**Fig. 2:**
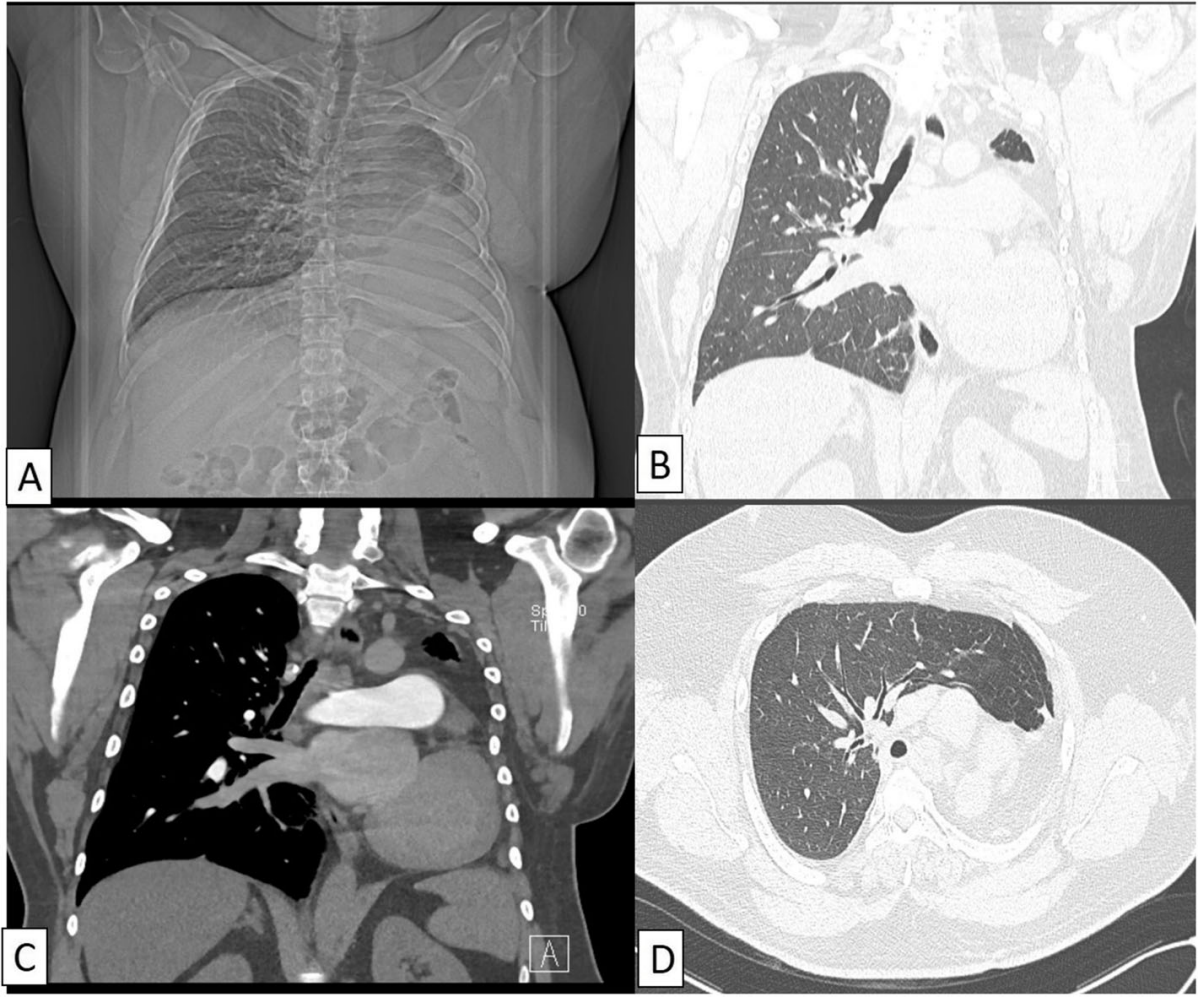
CT topogram (**A**) showing homogenous opacity of left hemithorax with non-bifurcation of trachea with mediastinal shift and contralateral herniation of right lung. CT images in coronal lung window (**B**), coronal mediastinal window (**C**), and axial lung window settings (**D**) showing absent left lung with shift of mediastinal structures into left hemithorax

**Fig. 3:**
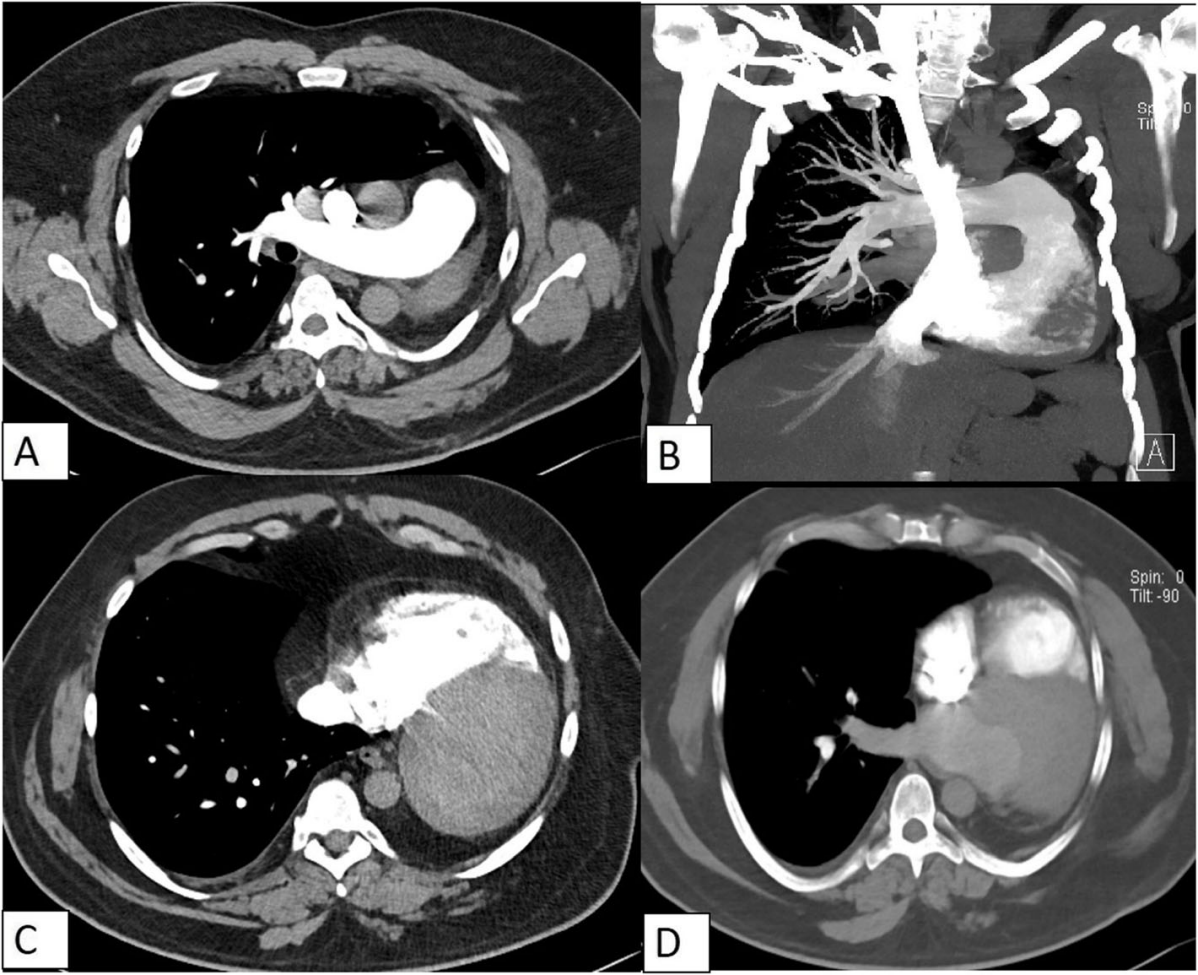
CT pulmonary angiogram showing continuation of patent main pulmonary artery into right pulmonary artery with absent left pulmonary artery (**A**, **B**). The heart is shifted into left hemithorax, lying flush with the chest wall with increased pericardial fat (**C**, **D**)

**Fig. 4:**
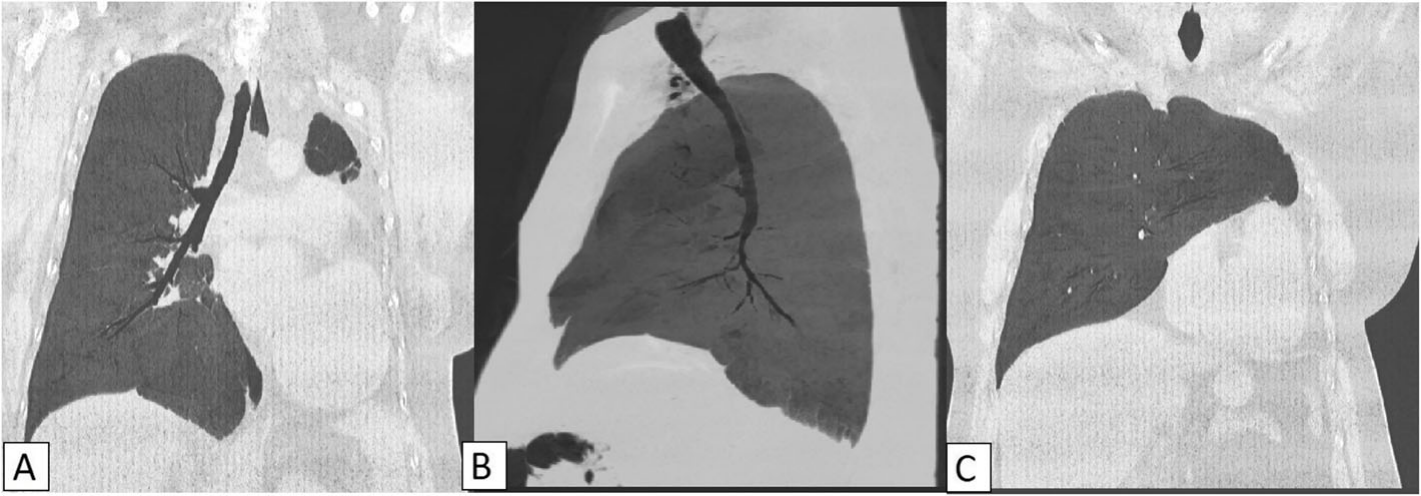
Minimum intensity projection CT images in coronal and sagittal planes show solitary right bronchus and lung (**A**, **B**). Mild tracheal narrowing is seen in its upper intrathoracic part (**B**). There is herniation of hypertrophied right lung across the midline into the left upper hemithorax (**C**)

CTPA demonstrated complete absence of left lung, left main bronchus, with absent left pulmonary artery as well as pulmonary veins (Fig. 2). Heart and mediastinal structures were situated in the left hemi-thorax (Fig. 2). The right lung was hypertrophied with contralateral herniation anteriorly into left hemithorax (Fig. 3). The trachea continued without bifurcation into right main bronchus and was mildly narrowed in upper intrathoracic part (Fig. 4). There was increased amount of fat in left hemithorax around the heart. Based on these findings, the diagnosis of unilateral left lung agenesis was made. No pneumonia or pleural effusion was seen on right side.

The patient was managed conservatively with oxygen and nebulization. Additional tests like echocardiography and ultrasound of abdomen were negative for any co-existing congenital cardiac or abdominal anomaly. On the day of discharge, patient was vitally stable, afebrile, doing well with no complaints of dyspnea. The existing clinical condition was explained to the patient and he was attached to out-patient department for follow-up and advised to report to the hospital in case of development of any symptoms related to respiratory system or fever.

## Discussion

In nearly half of the cases, unilateral lung agenesis is accompanied by other anomalies of the cardiovascular, gastrointestinal, musculoskeletal, or genitourinary systems. The commonest associated cardiac defects are tricuspid insufficiency, atrial and ventricular septal defects, patent ductus arteriosus, tetralogy of Fallot, aortic coarctation, and anomalous pulmonary venous return [5].

The lungs start developing toward the late first month of intrauterine life when a ventral bud (respiratory diverticulum) arises from the foregut which elongates and divides to form two lungs. The failure of division of this primitive bud into two leads to failure in the development of one lung whereas the other lung develops normally [6]. Depending on the stage of arrest of growth process, there can be complete or partial absence of lung tissue. It has been divided into 3 types, initially by Schneider and later refined by Boyden [7].

1. Pulmonary agenesis (type 1): There is complete absence of bronchus, lung tissue, and pulmonary vessels.
2. Pulmonary aplasia (type 2): There is a short, rudimentary, blind ending main bronchus with absent lung tissue and pulmonary vessels.
3. Pulmonary hypoplasia (type 3): There is presence of variable amounts of lung tissue, bronchial tree, and pulmonary vasculature.

However, from the functional point of view, unilateral lung agenesis and lung aplasia represent the same entity.

The exact etiology of pulmonary agenesis is not known. It has been postulated to occur secondary to a complex interplay of environmental insults, genetic, and mechanical factors [5].

The condition presents usually in the first few months or years of life and nearly half of the cases die in the first 5 years of life [5]. These children usually present with respiratory distress or recurrent chest infections. They are at an increased risk of pulmonary infections possibly due to the altered mechanics of trachea owing to its stretching and compressing due to the mediastinal shift. Late presentation is very uncommon. A total of 66 cases of unilateral lung agenesis presenting in adulthood have been reported till date. The oldest patient reported was 72 years old [8]. In some instances, the diagnosis is made accidentally from a radiograph or on autopsy. The timing of clinical presentation and the further clinical course is greatly modified by the presence of associated anomalies affecting the cardiovascular, musculoskeletal, gastrointestinal, or genitourinary systems. The condition is also associated with tracheal abnormalities like narrowing and stenosis [9, 10].

On clinical examination, the patient may have deformity of chest wall on the affected side or scoliosis. There may be reduced respiratory movements on inspection. On auscultation, breath sounds will be absent particularly at the base and axilla as this is occupied by the heart and other mediastinal structures. However, breath sound may be heard in the upper zone owing to the presence of herniated opposite lung into the upper part of affected hemithorax.

Before the availability of cross sectional imaging, the diagnosis was usually clinched at autopsy However, due to the advancements in diagnostic armamentarium, ante-mortem diagnosis of this anomaly has become easy.

Chest radiography reveals large basal homogenous opacity corresponding to the shifted heart and great mediastinal vascular structures with signs of volume loss reflected by the raised hemidiaphragm, and mediastinal shift. There is herniation of opposite hypertrophied lung with associated lucent opacity in the affected hemithorax in the upper zone. On radiograph, the differential diagnosis includes total lung collapse, diaphragmatic hernia, cystic adenomatoid lung malformations, pulmonary sequestration, scimitar syndrome, and prior pneumonectomy [11].

CT is an excellent modality as it provides exquisite details about the anatomy of tracheo-bronchial tree, lungs, heart, and major mediastinal vessels. CT shows direct evidence of continuation of trachea into a single bronchus with contralateral absent bronchus and lung. The main pulmonary artery also does not bifurcate and continues into a single pulmonary artery. The pulmonary veins on the affected side are absent. Heart and great mediastinal vessels are shifted into the affected hemithorax. The heart usually lies flush with the chest wall. There is variable degree of proliferation of fat on the affected side [12].

There is enlargement of the solitary lung. This enlargement represents true hypertrophy of the lung to meet the physiological demands of the body. It is essential to demonstrate and report any tracheal or bronchial narrowing or kinking due to the mediastinal shift. Any compression of mediastinal vessels is essential to report as these have therapeutic implications [4].

Due to the refinements in ultrasound and magnetic resonance imaging, the condition can be diagnosed antenatally [13].

Asymptomatic cases do not require any specific treatment apart from regular follow-up. However, earnest treatment is required for those presenting with lower respiratory tract infections. Some patients may be the candidates for corrective surgery to alleviate symptoms of trachea-bronchial narrowing or kinking or vascular compression. The procedures aimed at mediastinal stabilization include slide tracheoplasty, aortopexy, aortic resection and grafting, expansion prosthesis, and detour of the left pulmonary artery [14]. The prognosis of this condition is impacted by two factors of associated congenital malformations and the status of the normal lung. Any acquired affliction of the normal lung particularly infections can be life threatening and must be treated aggressively for a favorable outcome.

## Conclusion

Unilateral pulmonary agenesis is a rare congenital anomaly which commonly comes to clinical attention in infancy or early childhood. Presentation in late adulthood, as seen in the present case, is extremely rare. This case report highlights that, a rare condition like unilateral pulmonary agenesis, should be considered in the list of differentials in an adult presenting with opaque hemithorax with ipsilateral mediastinal shift on radiography.

## Data Availability

The data is available with the first three authors.

## Abbreviation

CTPA: Computed tomographic pulmonary angiography
